# Hypophosphatemia after CAR-T cell therapy: a prospective study of inflammation, tubular injury and phosphate handling

**DOI:** 10.1093/ckj/sfag307

**Published:** 2026-09-03

**Authors:** Francesc Moncho-Francés, Rafael Hernani, Miguel Angel Solis-Salguero, Ana Benzaquén, Isabel Juan-García, Ariadna Pérez-Martínez, Mireia Micó, Juan F Navarro-González, Jose Luis Piñana, Juan Carlos Hernández Boluda, María Jose Terol, Carlos Solano, Jose Luis Górriz, Isidro Torregrosa

**Affiliations:** Nephrology Department, Hospital Clínico Universitario de Valencia, INCLIVA Biomedical Research Institute, Valencia, Spain; Hematology Department, Hospital Clínico Universitario de Valencia, INCLIVA Biomedical Research Institute, Valencia, Spain; Nephrology Department, Hospital Clínico Universitario de Valencia, INCLIVA Biomedical Research Institute, Valencia, Spain; Hematology Department, Hospital Clínico Universitario de Valencia, INCLIVA Biomedical Research Institute, Valencia, Spain; Nephrology Department, Hospital Clínico Universitario de Valencia, INCLIVA Biomedical Research Institute, Valencia, Spain; Hematology Department, Hospital Clínico Universitario de Valencia, INCLIVA Biomedical Research Institute, Valencia, Spain; Hematology Department, Hospital Clínico Universitario de Valencia, INCLIVA Biomedical Research Institute, Valencia, Spain; Research Unit and Nephrology Department, Hospital Universitario Nuestra Señora de Candelaria, Santa Cruz de Tenerife, Spain; Institute of Biomedical Technologies, Universidad de La Laguna, La Laguna, Santa Cruz de Tenerife, Spain; Faculty of Health Sciences, Universidad Fernando Pessoa Canarias, Las Palmas de Gran Canaria, Spain; RICORS2040-RENAL (RD24/0004/0022), Instituto de Salud Carlos III, Madrid, Spain; Hematology Department, Hospital Clínico Universitario de Valencia, INCLIVA Biomedical Research Institute, Valencia, Spain; Hematology Department, Hospital Clínico Universitario de Valencia, INCLIVA Biomedical Research Institute, Valencia, Spain; Department of Medicine, Faculty of Medicine, Universitat de València, Valencia, Spain; Hematology Department, Hospital Clínico Universitario de Valencia, INCLIVA Biomedical Research Institute, Valencia, Spain; Department of Medicine, Faculty of Medicine, Universitat de València, Valencia, Spain; Hematology Department, Hospital Clínico Universitario de Valencia, INCLIVA Biomedical Research Institute, Valencia, Spain; Department of Medicine, Faculty of Medicine, Universitat de València, Valencia, Spain; Nephrology Department, Hospital Clínico Universitario de Valencia, INCLIVA Biomedical Research Institute, Valencia, Spain; RICORS2040-RENAL (RD24/0004/0022), Instituto de Salud Carlos III, Madrid, Spain; Department of Medicine, Faculty of Medicine, Universitat de València, Valencia, Spain; Nephrology Department, Hospital Clínico Universitario de Valencia, INCLIVA Biomedical Research Institute, Valencia, Spain

**Keywords:** CAR-T therapy, electrolyte disturbances, hypophosphatemia, inflammation, tubular injury

## Abstract

**Background:**

Hypophosphatemia is a frequent electrolyte abnormality after chimeric antigen receptor T-cell (CAR-T) therapy, but the clinical and biological factors associated with phosphate decline remain poorly defined. We prospectively evaluated the incidence, determinants, and potential biological associations of hypophosphatemia after CAR-T therapy.

**Methods:**

We conducted a prospective study including 63 adults with B-cell lymphoma treated with CAR-T therapy between June 2020 and December 2023. Serum phosphate was measured daily during hospitalization. First-morning urine and blood samples were obtained at infusion (D0) and on days +7 (D7) and +14 (D14). Fractional excretion of phosphate (FEPO₄), urine protein-to-creatinine ratio (uPCR), parathyroid hormone (PTH), and fibroblast growth factor 23 (FGF23) were assessed. Patients were stratified according to the presence of moderate hypophosphatemia.

**Results:**

Hypophosphatemia occurred in 49/63 patients (78%), including 26 (41%) with moderate hypophosphatemia. Median time to phosphate nadir was 6 days. Patients with moderate hypophosphatemia had higher baseline lactate dehydrogenase levels and Endothelial Activation and Stress Index scores, lower phosphate and creatinine levels at infusion, and a higher frequency of prior ifosfamide exposure. Higher uPCR at D7, higher peak interleukin-6 levels and higher FEPO₄ at D7 were associated with lower phosphate nadir values (*ρ* = −0.55, *P* < .001; *ρ* = −0.55, *P* < .001; and *ρ* = −0.31, *P* = .018, respectively). FGF23 levels were not associated with hypophosphatemia in the primary analysis, while PTH showed a modest inverse association with phosphate nadir in exploratory analysis. Moderate hypophosphatemia was associated with inferior overall survival in univariable analysis.

**Conclusions:**

Hypophosphatemia is common after CAR-T therapy and was associated with systemic inflammation, urinary markers compatible with partial proximal tubular dysfunction and altered renal phosphate handling. Secondary hyperparathyroidism may have contributed in a subset of patients, whereas FGF23 did not appear to be a dominant explanatory pathway in this cohort.

## Graphical Abstract

**Graphical Abstract:**
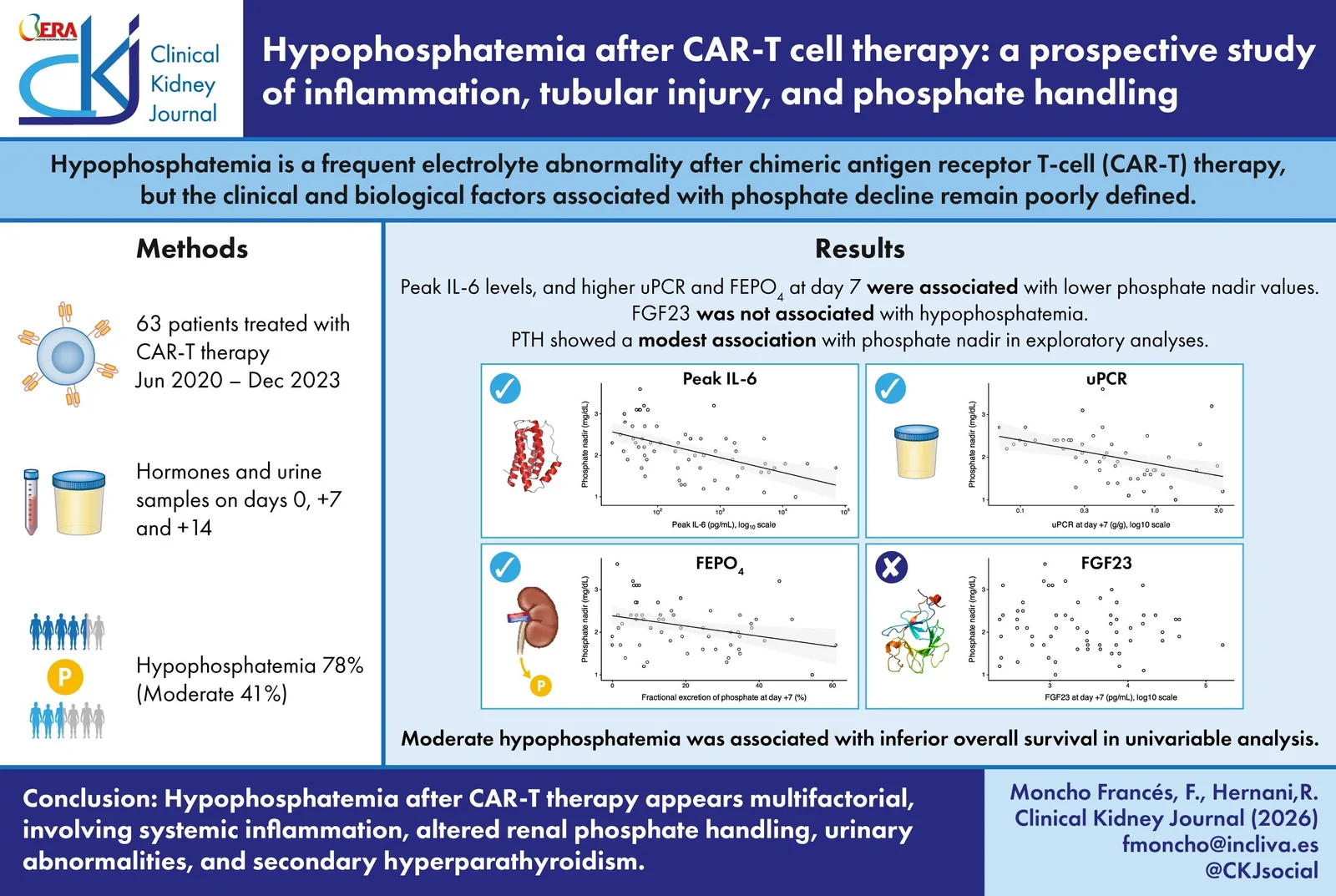

KEY LEARNING POINTS **What was known:**

- Hypophosphatemia is one of the most frequent electrolyte abnormalities after CAR-T cell therapy and has been associated with CRS and ICANS.
- The mechanisms underlying phosphate decline after CAR-T therapy have remained unclear and are likely multifactorial.
- Prospective data evaluating renal phosphate handling and tubular injury in this setting have been limited.

**This study adds:**

- Hypophosphatemia occurred in 78% of patients after CAR-T therapy, with 41% developing moderate hypophosphatemia.
- Lower phosphate nadir was associated with higher IL-6 levels, higher proteinuria, and higher FEPO₄, supporting a link between inflammation, tubular injury, and altered renal phosphate handling.
- FGF23 was not associated with hypophosphatemia, whereas PTH showed a modest association with phosphate nadir in exploratory analyses, suggesting that secondary hyperparathyroidism may contribute to the development of hypophosphatemia.

**Potential impact:**

- Hypophosphatemia after CAR-T therapy may reflect a multifactorial process, involving systemic inflammation, urinary abnormalities, partial proximal tubular dysfunction, and secondary hyperparathyroidism.
- Serum phosphate is inexpensive and routinely available and may serve as a practical biomarker of early toxicity burden after CAR-T therapy.
- Monitoring phosphate together with urinary abnormalities may improve early identification of patients at higher risk of therapy-related complications.

## INTRODUCTION

Chimeric antigen receptor T-cell (CAR-T) therapy has transformed the management of relapsed or refractory lymphoid haematological malignancies [1]. CAR-T cell indications are rapidly expanding beyond cancer to autoimmune diseases [2] and infectious conditions [3].

Despite its efficacy, CAR-T therapy is associated with a distinctive toxicity profile driven by immune and metabolic activation. Besides cytokine release syndrome (CRS) and immune effector cell-associated neurotoxicity syndrome (ICANS) [4], renal complications are increasingly recognized, ranging from acute kidney injury to electrolyte abnormalities [5].

Hypophosphatemia has emerged as one of the most frequent electrolyte abnormalities after infusion [6–9]. Lower serum phosphate levels have been associated with both occurrence and severity of CRS [10, 11] and ICANS [7, 12]. The mechanisms responsible for phosphate decline after CAR-T therapy remain incompletely understood and are likely multifactorial. Experimental data suggest that activated CAR-T cells increase phosphate consumption to sustain the high metabolic demands of effector function [7] while limited clinical evidence supports a contribution of renal phosphate wasting [10].

Fibroblast growth factor 23 (FGF23), a major regulator of phosphate homeostasis, has been implicated as a potential mediator of hypophosphatemia in inflammatory states [13]. Given that CAR-T therapy is characterized by intense cytokine activation, FGF23 represents a biologically plausible contributor to post-infusion phosphate decline through increased urinary phosphate losses [6].

In this prospective study, we aimed to characterize the incidence and determinants of hypophosphatemia after CAR-T therapy, with particular emphasis on urinary phosphate losses, biomarkers of tubular injury, mineral metabolism parameters including FGF23 and PTH, and inflammatory parameters.

## MATERIALS AND METHODS

### Study population

A prospective, single-centre study was conducted including all adult patients with B-cell lymphoma treated with CAR-T therapy at Hospital Clínico Universitario de Valencia from 1 June 2020 to 31 December 2023. During hospitalization, routine laboratory tests were collected. In addition, plasma samples for PTH, FGF23, and first morning void urine samples were obtained on days 0 (D0), +7 (D7), and +14 (D14). Values below the lower limit of quantification were imputed assuming normal distribution. After discharge, follow-up was limited to disease progression and mortality until December 2024. All protocols complied with the ethical standards of the Declaration of Helsinki were reviewed and approved by the Clinical Ethics Committee of the Hospital Clínico Universitario de Valencia, and informed consent was obtained from all patients (reference number: 2021/220).

Clinical data were retrieved from the electronic health records. These included demographic information, hematologic malignancy type, chemotherapy treatments, prognostic risk stratification, and patients’ comorbidities such as diabetes mellitus, hypertension, and chronic kidney disease (CKD). Kidney function was assessed using estimated glomerular filtration rate (eGFR), calculated using the CKD-EPI 2021 equation [14].

### Definitions

Baseline laboratory values were those obtained at CAR-T infusion, unless otherwise specified. Serum phosphate levels were measured daily during hospitalization. Phosphate nadir was defined as the lowest serum phosphate value recorded within the first 15 days after CAR-T infusion.

For the primary analyses, patients were stratified into two groups based on moderate hypophosphatemia, defined as a phosphate nadir below 2.0 mg/dl, and compared with patients with mild or no hypophosphatemia. Fractional excretion of phosphate (FEPO_4_) was calculated using a standard formula based on paired serum and urine phosphate and creatinine concentrations. FEPO₄ >5% was considered suggestive of inappropriate renal phosphate loss in the context of hypophosphatemia [15]. Urinary protein excretion was assessed using urine protein-to-creatinine ratio (uPCR), expressed in g/g, from urine samples collected at D0, D7, and D14. Urinary alpha-1-microglobulin-to-creatinine ratio (uA1M/Cr) was used as an additional urinary marker compatible with tubular dysfunction. Urine dipstick testing was reviewed for glycosuria.

Parathyroid hormone (PTH) was measured using a second-generation intact PTH immunoassay and interpreted according to the laboratory-certified reference range 25–120 pg/ml. Intact FGF23 and interleukin-6 (IL-6) were measured as described in the Supplementary Material.

CRS and ICANS were graded according to the American Society for Transplantation and Cellular Therapy recommendations [16] and managed following institutional guidelines [17]. Severe forms of CRS or ICANS were defined as grade 3 or higher. Overall response rate was defined as the proportion of patients who achieved either partial (PR) or complete response (CR) after CAR-T infusion [18]. The Endothelial Activation and Stress Index (EASIX) score was calculated prior to lymphodepleting chemotherapy, as previously described [19]. The International Prognostic Index (IPI) was calculated based on five clinical factors: age >60 years, stage III/IV disease, elevated lactate dehydrogenase (LDH), ECOG performance status ≥2, and >1 extranodal site [20].

### Statistical analysis

Categorical variables were expressed as numbers and percentages, and continuous variables as mean *± * SD or median with interquartile range (IQR). Between-group comparisons, correlation analyses, multivariable logistic regression, and survival analyses were performed as appropriate. Given the limited sample size, multivariable models were considered exploratory. A two-sided *P*-value <.05 was considered statistically significant. Full details of the statistical analysis are provided in the Supplementary Materials.

## RESULTS

Sixty-three patients received CAR-T therapy during the study period: axicabtagene ciloleucel (*n* = 47; 75%), tisagenlecleucel (*n* = 10; 16%), and a 4-1BB-based investigational product (*n* = 6; 9%). Diffuse large B-cell lymphoma was the underlying diagnosis in 90% of cases, and all patients had relapsed or refractory disease after at least two prior treatment lines. Lymphodepletion consisted mainly of fludarabine plus cyclophosphamide (98%), with dose adjustment required in seven patients due to CKD.

During follow-up, hypophosphatemia occurred in 49 patients (78%): mild in 23 patients (37%) and moderate in 26 (41%). No cases of severe hypophosphatemia were observed. The median time to phosphate nadir was 6 days (IQR, 3–8). At the time of CAR-T infusion, 12 patients (20%) already had hypophosphatemia, although this was moderate in only two (3%). After CAR-T infusion, *de novo* hypophosphatemia developed in 39 patients, while 10 of the 12 patients with baseline hypophosphatemia showed phosphate decline. Two patients (3%) presented gastro-intestinal losses, while phosphate supplementation was administered in 30 patients (48%) according to clinical judgement. By day 15, hypophosphatemia persisted in 10 patients (16%), with moderate hypophosphatemia present in three (5%).

Baseline characteristics stratified by the occurrence of moderate hypophosphatemia (nadir <2 mg/dl) are shown in Table 1. Patients who developed moderate hypophosphatemia had higher baseline eGFR, LDH levels and EASIX score, lower phosphate levels at infusion, and a higher frequency of prior ifosfamide exposure, whereas other baseline characteristics were similar between groups.

**Table 1:** Baseline characteristics.

	*N* = 63	Phosphorus nadir levels (mg/dl)		*P*
		< 2 (*n* = 26)	> 2 (*n* = 37)	
Age in years, median (IQR)	62 (49–68)	57 (42–67)	62 (54–69)	0.12
Male sex, *n* (%)	35 (56)	10 (39)	25 (68)	0.02
Hypertension, *n* (%)	18 (28)	4 (15)	14 (38)	0.05
Diabetes, *n* (%)	9 (14)	3 (12)	6 (16)	0.72
CKD, *n* (%)	7 (11)	3 (12)	4 (11)	0.43
Previous treatment with cisplatin, *n* (%)	47 (75)	21 (81)	26 (70)	0.21
Previous treatment with ifosfamide, *n* (%)	28 (44)	16 (62)	12 (32)	0.014
eGFR at infusion in ml/min/1.73 m^2^	104.0 (88.0–112.6)	109.1 (97.6–118.4)	101.7 (83.1–109.7)	0.04
Phosphorus at infusion in mg/dL	3.1 (2.7–3.5)	2.9 (2.3–3.1)	3.3 (3.0–3.6)	< 0.001
LDH preLD in U/L, median (IQR)	548 (355–1088)	1214 (546–1938)	428 (255–576)	< 0.001
EASIX score preLD, median (IQR)	2.54 (1.48–6.40)	5.25 (2.06–10.5)	2.17 (1.21–3.99)	0.004
Diagnosis, *n* (%)				0.22
DLBCL	57 (90)	22 (85)	35 (95)	
PMBCL	6 (10)	4 (15)	2 (5)	
IPI preLD *≥ * 3, *n* (%)	31 (49)	14 (54)	17 (46)	0.54
Ann Arbor stage preLD III–IV, *n* (%)^a^	41 (68)	17 (68)	24 (69)	0.96
Disease status preLD, *n* (%)^a^				0.02
Disease progression	41 (68)	23 (88)	18 (53)	
Stable disease	6 (10)	1 (4)	5 (15)	
Partial response	7 (12)	0 (0)	7 (20)	
Complete response	6 (10)	2 (8)	4 (12)	
CAR-T cell construct, *n* (%)				0.05
Tisagenlecleucel	10 (16)	3 (12)	7 (19)	
Axicabtagene ciloleucel	47 (75)	23 (88)	24 (65)	
Investigational product 4-1BB	6 (9)	0 (0)	6 (16)	

CKD, chronic kidney disease; eGFR: estimated glomerular filtration rate, LDH, lactate dehydrogenase; EASIX, endothelial activation and stress index; DLBCL, diffuse large B-cell lymphoma; PMBCL, primary mediastinal B-cell lymphoma; preLD: pre-lymphodepletion; IPI, international prognostic index.

^a^Disease status at LD and stage was not assessed in three patients.

Among patients with CKD, four developed hypophosphatemia during follow-up. Although baseline eGFR was not significantly associated with hypophosphatemia, median eGFR was numerically higher in patients who developed hypophosphatemia than in those who did not (114 vs. 60 ml/min/1.73 m², respectively).

### Factors associated with hypophosphatemia in CAR-T cell therapy

Inflammatory markers were significantly higher in patients who developed moderate hypophosphatemia. Both C-reactive protein (CRP) and peak IL-6 levels were increased in this group. Median time to peak IL-6 was 5 days (IQR, 4–7) while median time to phosphate nadir was 6 days (IQR, 3–8). We found a consistent inverse correlation between peak IL-6 levels and phosphate nadir (*ρ* = −0.55, *P* < .001), supporting a close association between systemic inflammation and phosphate decline (Fig. 1A).

**Figure 1:**
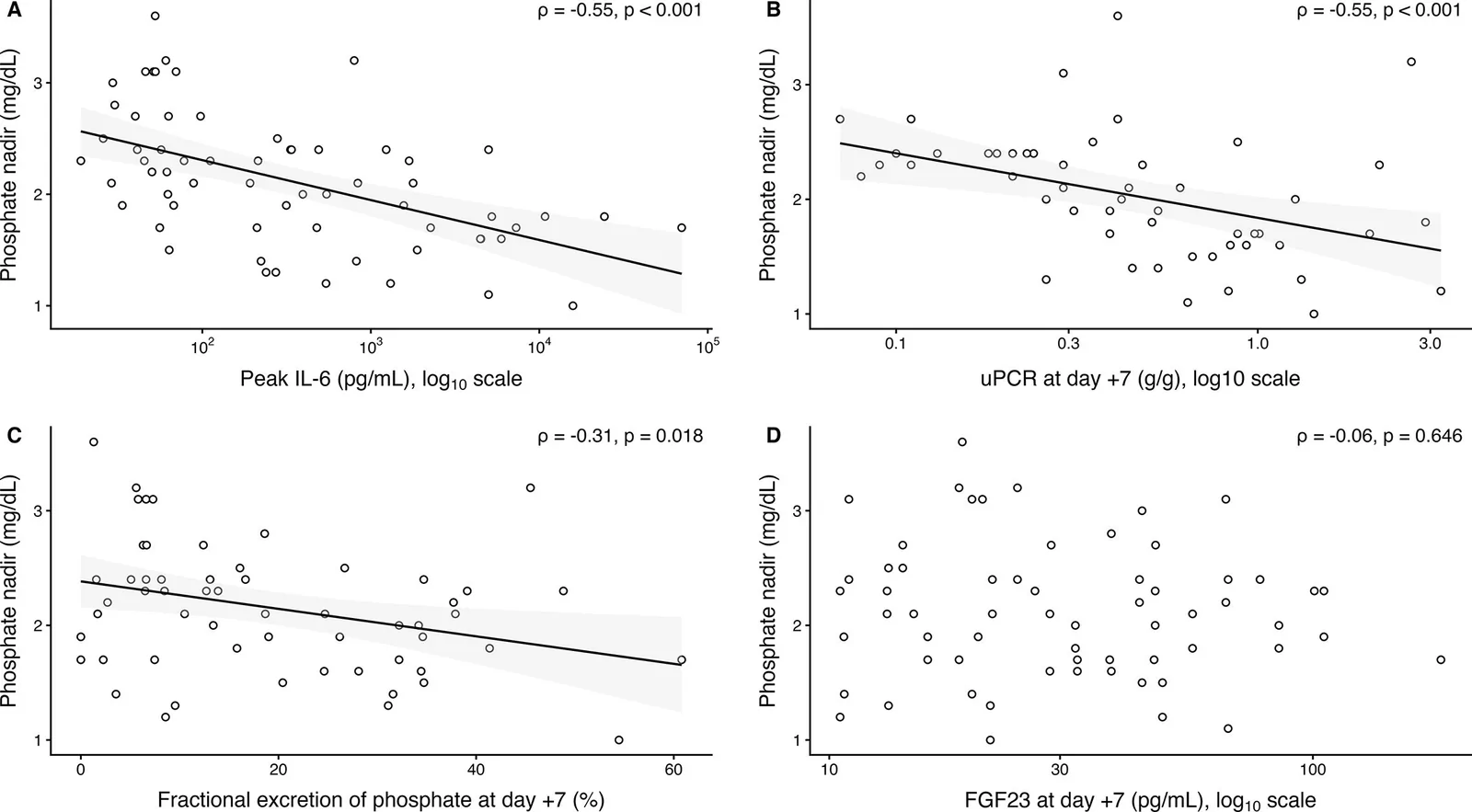
(A) Inverse association between peak IL-6 levels and phosphate nadir. IL-6 values are shown on a log 10 scale (Spearman *ρ* = −0.55; *P* < .001). (B) Inverse association between uPCR at D7 and phosphate nadir. uPCR values are shown on a log 10 scale (Spearman *ρ* = −0.55; *P* < .001). (C) Inverse association between fractional excretion of phosphate (FEPO₄) at D7 and phosphate nadir. Higher urinary phosphate excretion was associated with lower phosphate levels (Spearman *ρ* = −0.31; *P* = .018). (D) No significant association between FGF23 levels at D7 and phosphate nadir. FGF23 values are shown on a log 10 scale (Spearman *ρ* = −0.06; *P* = .646).

Patients with moderate hypophosphatemia exhibited higher uPCR and uA1M/Cr at D7. No glycosuria was detected in any patient. uPCR and uA1M/Cr were inversely correlated with phosphate nadir [Spearman *ρ* = −0.55, *P* < .001 (Fig. 1B) and *ρ* = −0.43, *P* = .001, respectively].

FEPO_4_ at D7 was numerically higher in patients with moderate hypophosphatemia, although without reaching statistical significance (*P* = .13). Notably, 18 patients (69%) in the moderate hypophosphatemia group had FEPO_4_ values >5%, suggestive of inappropriate renal phosphate loss. Moreover, FEPO_4_ was inversely correlated with phosphate nadir (Spearman *ρ* = −0.31, *P* = .018), suggesting a possible contribution of altered renal phosphate handling during the early post-CAR-T period (Fig. 1C).

Longitudinal analysis of FGF23 concentrations did not reveal significant between-group differences across time points (Fig. 1D and Supplementary Figures S1 and S2). Although PTH was not associated with hypophosphatemia in the primary between-group analysis, PTH levels tended to be higher in patients with moderate hypophosphatemia. In an exploratory analysis excluding two patients with baseline eGFR <60 ml/min/1.73 m², PTH showed a modest inverse correlation with phosphate nadir (*ρ* = -0.26, *P* = .045) (Supplementary Figure S3). Baseline 25-hydroxyvitamin D levels were lower in patients with moderate hypophosphatemia. Inflammatory, urinary and hormonal parameters stratified by hypophosphatemia severity are summarized in Table 2.

**Table 2:** Inflammatory, tubular, and hormonal parameters according to hypophosphatemia severity.

	*N* = 63	Phosphorus nadir levels (mg/dl)		*P*
		< 2 (*n* = 26)	> 2 (*n* = 37)	
CRP maximum in mg/L	148 (76–273)	236 (147–334)	105 (48–209)	<0.001
IL-6 maximum in pg/mL	239 (56–1568)	1439 (235–5406)	70 (48–366)	<0.001
uPCR at day 7 in g/g	0.4 (0.1–0.8)	0.8 (0.4–1.1)	0.2 (0.1–0.4)	<0.001
FePO_4_ at day 7 in %	15 (6–32)	25 (8–35)	12 (6–26)	0.13
uA1M/Cr at day 7 in mg/g	90.6 (35.3–186.3)	166.5 (84.3–263.6)	52.4 (19.6–142.8)	0.001
PTH at infusion in pg/mL	57 (41–90)	70 (41–103)	57 (41–80)	0.40
PTH at day +7 in pg/mL	65 (41–114)	77 (41–168)	62 (41–87)	0.17
25-OH Vitamin D at infusion in ng/mL	13 (9–22)	10 (7–21)	14 (11–23)	0.02
FGF23 at infusion in pg/mL	26.3 (21.6–38.7)	27.8 (20.1–38.9)	24.8 (21.7–41.0)	0.89
FGF23 at day 7 in pg/mL	28.7 (18.6–48.9)	32.4 (17.9–48.9)	28.6 (16.8–51.8)	0.98

CRP, C-reactive protein; IL-6, interleukin-6; uPCR, urine protein-to-creatinine ratio; uA1M/Cr, urinary alpha-1-microglobulin-to-creatinine ratio; FEPO₄, fractional excretion of phosphate; PTH, parathyroid hormone; FGF23, fibroblast growth factor 23. Values are expressed as median [interquartile range (IQR)].

CAR-T cell expansion at D7 was available for 51 patients. Expansion levels did not differ between patients with moderate hypophosphatemia and those with mild or no hypophosphatemia [log 10-transformed values: 1.48 (IQR, 1.23–1.77) vs. 1.37 (IQR, 0.34–2.24), *P* = .91]. In addition, no correlation was observed between absolute CAR-T cell counts at D7 and phosphate nadir. Phosphate nadir differed across CAR-T constructs overall (*P* = .02), with median values of 2.0 mg/dl (IQR 1.7–2.4) for axicabtagene ciloleucel, 2.3 mg/dl (IQR 1.8–2.4) for tisagenlecleucel, and 3.05 mg/dl (IQR 2.2–3.1) for the investigational product, whereas no difference was observed between axicabtagene ciloleucel and tisagenlecleucel (*P* = .40).

In exploratory partial correlation analyses, the inverse association between phosphate nadir and log-transformed IL-6 remained robust after adjustment for PTH (*ρ* = -0.53, *P* < .001), whereas the inverse association between phosphate nadir and PTH after adjustment for IL-6 was weaker and no longer statistically significant (*ρ* = -0.25, *P* = .06). After adjustment for IL-6, the associations of phosphate nadir with uPCR and FEPO₄ were also attenuated and no longer statistically significant. However, IL-6 remained positively correlated with both uPCR (*ρ* = 0.646, *P* < .001) and FEPO₄ (*ρ* = 0.348, *P* = .008), and uPCR was also positively correlated with FEPO₄ (*ρ* = 0.649, *P* < .001). Overall, these exploratory findings suggest that hypophosphatemia was more closely associated with inflammatory burden than with PTH.

### Other electrolyte disturbances

Electrolyte disturbances were common after CAR-T infusion, occurring in 95% of patients, with moderate disorders observed in 51% and severe disturbances in only one case. Hypophosphatemia, as described, was the most frequent abnormality (78%), followed by hypokalaemia (70%) and hyponatremia (59%), with electrolyte nadirs occurring at a median of 5 days (IQR, 4–8) after infusion.

No association was observed between phosphate levels and sodium, magnesium, or calcium. In contrast, hypophosphatemia was associated with hypokalaemia (Supplementary Table S1), and lower phosphate levels correlated with lower potassium levels (Spearman *ρ* = 0.33, *P* = .009). Patients with lower potassium levels also had higher urinary potassium-to-creatinine ratio at D7 (Supplementary Figure S4).

### CAR-T therapy related complications, response, progression, and survival

Fifty-three (87%) patients developed CRS and 32 (52%) ICANS. Grade ≥3 CRS and ICANS occurred in 6 (10%) and 15 (26%) patients, respectively. The median time from CAR-T infusion to CRS and ICANS was 1 day (IQR, 1–3) and 7 days (IQR, 5–8), respectively.

No significant differences in the incidence or severity of CRS or ICANS were observed according to hypophosphatemia severity. However, any grade of hypophosphatemia was associated with a higher incidence of ICANS, occurring in 59% of patients with hypophosphatemia compared with 29% of those without (*P* = .04).

Forty-six patients (73%) achieved an objective response, including 35 (56%) CR and 11 (17%) PR, at a median time of 37 days (IQR, 33–44) after CAR-T infusion. During follow-up, 34 patients (54%) experienced disease progression, including 15 (24%) with refractory disease and 19 (30%) with relapse. The median progression-free survival was 242 days (IQR, 56–503). Response rates differed according to hypophosphatemia severity (*P* = .029). Patients with moderate hypophosphatemia achieved CR less frequently than those with mild or no hypophosphatemia (38% vs. 68%).

Thirty-five patients (56%) died at a median of 153 days (IQR, 91–284) after CAR-T infusion. The primary causes of death were relapsed or refractory disease [R/R] (*n* = 28, 80%), infection (*n* = 5, 14%), and ICANS (*n* = 2, 6%).

During the first year after CAR-T, patients who developed moderate hypophosphatemia exhibited lower overall survival compared with those with mild or no hypophosphatemia (Fig. 2). In univariable Cox regression analysis, moderate hypophosphatemia was associated with a two-fold increased risk of death (HR 2.11, 95% CI 1.01–4.39, *P* = .046). In an adjusted Cox model including age and EASIX, the association was attenuated and did not retain statistical significance, whereas EASIX remained independently associated with mortality.

**Figure 2:**
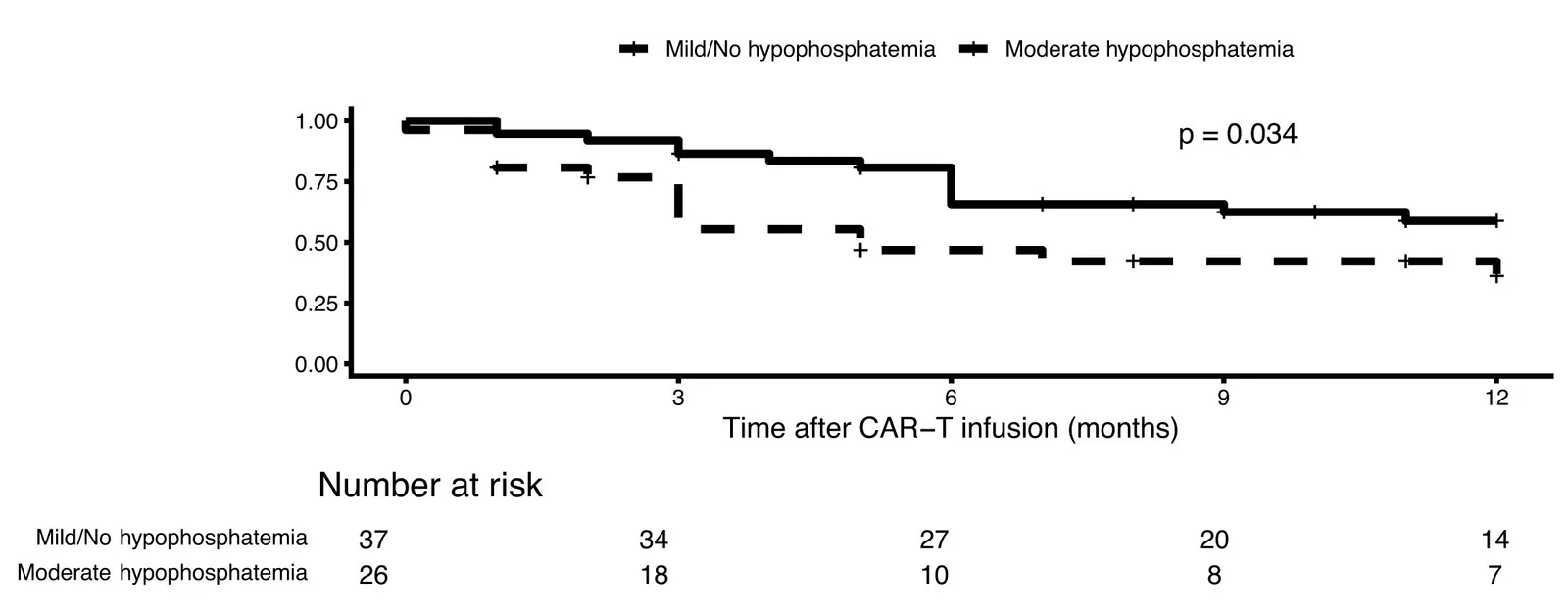
Overall survival according to hypophosphatemia severity. Kaplan–Meier analysis stratified by moderate hypophosphatemia versus mild or no hypophosphatemia (log-rank *P* = .034).

## DISCUSSION

Hypophosphatemia was the most frequent electrolyte disturbance after CAR-T cell therapy in our cohort. Lower phosphate levels were associated with systemic inflammation, urinary abnormalities compatible with partial tubular dysfunction, and altered renal phosphate handling. Hypophosphatemia during CAR-T therapy may represent a marker of disease severity and systemic inflammation and could be associated with worse clinical outcomes in these patients.

Previous studies described a high incidence of electrolyte disturbances, identifying hypophosphatemia as the most common abnormality, attributed to redistribution or increased metabolic activity [6, 7, 21, 22]. This mechanism may be particularly relevant in the early post-infusion period and cannot be excluded by our data. However, no association was observed between CAR-T cell expansion and hypophosphatemia, suggesting a limited role for increased cellular phosphate consumption itself.

In our cohort, the correlation and close temporal relationship between peak IL-6 and phosphate nadir is compatible with an acute inflammation-related process. Peak IL-6 levels were inversely correlated with phosphate nadir, while urinary markers (including uPCR and uA1M/Cr) were significantly elevated. These findings are compatible with transient tubular dysfunction and altered phosphate handling in the context of systemic inflammation [5, 9, 23]. The proximal tubule is particularly susceptible to inflammatory injury, and transient dysfunction of this segment may explain the temporal pattern of hypophosphatemia observed after CAR-T infusion [24–26]. However, the absence of glycosuria and the inability to assess acid–base status, aminoaciduria or urate handling preclude the diagnosis of complete proximal tubular syndrome. These findings were in line with previous work by Nakamura *et al*. [10]. In addition, prior exposure to nephrotoxic agents such as ifosfamide may have influenced the risk of hypophosphatemia by predisposing to underlying tubular dysfunction, although assessment of this effect was beyond the scope of our study design [27].

Although inflammation-induced increases in FGF23 have been proposed as a potential mechanism of phosphaturia, our data do not support a major role for this pathway [13]. We found no association between FGF23 levels, hypophosphatemia and FEPO_4_. Of note, normal or non-suppressed FGF23 concentrations in the setting of hypophosphatemia may be considered physiologically inappropriate. However, the absence of an association between FGF23, phosphate nadir, and FEPO₄ suggests that FGF23 was unlikely to be the dominant explanatory pathway in this cohort. Nakamura *et al*. [10] reported similar hormonal observations in a subset of 10 patients, showing that post-CAR-T hypophosphatemia was not explained by FGF23 or PTH. In our cohort, PTH was not associated with hypophosphatemia in the primary between-group analysis, although an exploratory analysis excluding patients with eGFR <60 ml/min/1.73 m² showed a modest inverse correlation between PTH and phosphate nadir. This finding suggests that secondary hyperparathyroidism in response to low calcium levels may contribute in selected patients, but it did not appear to be the dominant factor in the overall cohort. While patients with moderate hypophosphatemia showed a trend toward lower 25-hydroxyvitamin D levels, vitamin D deficiency was highly prevalent overall, with 70% of patients presenting concentrations below 20 ng/ml, and no significant differences in hypophosphatemia were observed according to vitamin D status. Taken together, these findings suggest that classical phosphaturic hormonal pathways may not be the dominant drivers of hypophosphatemia consistent with those reported in critically ill patients, in whom renal phosphate wasting is frequent but not explained by FGF23 or other classical phosphaturic hormones [28].

Differences in hypophosphatemia across CAR-T constructs may reflect the distinct toxicity profiles of these products, particularly their differential inflammatory burden and expansion kinetics [29]. This finding should nevertheless be interpreted cautiously, as peak expansion may vary across CAR-T constructs [30].

Beyond its biological interpretation, hypophosphatemia may also reflect underlying disease and inflammatory burden. Patients who developed moderate hypophosphatemia exhibited higher baseline LDH and EASIX scores. In this context, serum phosphate may be particularly useful as a biomarker because it is inexpensive, routinely available, and easy to monitor in daily clinical practice [11].

Hypophosphatemia may also have specific clinical relevance in the context of neurotoxicity. In our cohort, hypophosphatemia <2.5 mg/dl was more frequent in patients who developed ICANS. Similar observations have been reported in retrospective series [7, 12], which further suggested that phosphorus repletion may reduce both the incidence and duration of ICANS [31]. Whether phosphate repletion can modify outcomes remains an important question for prospective investigation.

Our study has several limitations. First, it was conducted in a single centred and included a relatively limited sample size, which may restrict the generalisability of the findings. Second, urinary abnormalities were assessed at predefined time points rather than continuously, which may have limited the characterization of their temporal evolution and their relationship with serum phosphate changes. Moreover, urine and blood sampling did not necessarily coincide with the individual phosphate nadir. Third, FEPO₄ should be interpreted cautiously in this acute setting because it depends on serum phosphate concentrations and may be affected by rapid changes in circulating phosphate levels or intracellular phosphate redistribution. Fourth, we did not measure ionized calcium or other phosphaturic mediators that can contribute to hypophosphatemia. Fifth, interpretation of PTH is limited by the method-dependent variability of second-generation intact PTH assays; however, PTH was analysed as a continuous variable, and not solely according to a categorical threshold. Sixth, after discharge, follow-up was focused on disease progression and mortality, and long-term kidney function or persistent urinary abnormalities were not systematically assessed. Finally, the lack of systematic data on supportive care interventions, including intravenous fluids, phosphate replacement, vasopressors, and nephrotoxic medications is limiting, as these measures may have influenced the incidence and severity of electrolyte disturbances. Nevertheless, the prospective design and systematic assessment of phosphate levels, inflammatory parameters, and selected urinary indices provide novel insight into the mechanisms of hypophosphatemia after CAR-T therapy.

## CONCLUSION

Hypophosphatemia is a common complication after CAR-T therapy. Lower phosphate levels were associated with systemic inflammation, urinary abnormalities compatible with partial tubular dysfunction, and altered renal phosphate handling. These findings suggest that hypophosphatemia may reflect a multifactorial process and may serve as a simple and readily available biomarker of inflammatory burden and neurotoxicity risk. Prospective studies are warranted to clarify the clinical impact of phosphate correction in this setting.

## Supplementary Material

sfag307_Supplemental_File

## Data Availability

The data underlying this article are fully available within the published manuscript and the supplementary material.
