# Supplementary material for: Hypophosphatemia after CAR-T cell therapy: a prospective study of inflammation, tubular injury and phosphate handling
Source: Clin Kidney J. 2026 Sep 3;19(10):sfag307. doi: 10.1093/ckj/sfag307 (PMC13639187; doi:10.1093/ckj/sfag307)
Supplement: Supplementary file 1 [file sfag307_supplemental_file.docx]

**SUPPLEMENTARY METHODS**

**Laboratory measurements**

PTH was measured using the Abbott second-generation intact PTH immunoassay, which detects PTH 1–84 but may also recognize long PTH fragments, such as PTH 7–84. Results were interpreted according to the certified local laboratory reference interval of 25–120 pg/mL and with caution in patients with reduced kidney function. PTH was analyzed as a continuous variable rather than categorized according to a predefined threshold. Because PTH concentrations are method-dependent and differ across commercially available second-generation assays, interpretation was based on the analytical method used at our institution. The use of local reference intervals for the Abbott intact PTH assay is supported by recently published reference interval data [1].

For FGF23 assessment, blood samples were drawn in the morning after an 8-hour fast. Serum was aliquoted and immediately stored at −80°C until analysis. Intact FGF23 was measured using the Human FGF23 ELISA Kit (EMD Millipore Corporation, Milford, MA, USA), with a sensitivity of 3.5 pg/mL and intra- and inter-assay coefficients of variation of 9.5% and 6.85%, respectively. Values below the lower limit of quantification were imputed assuming a normal distribution.

IL-6 was measured as part of routine clinical monitoring. Peak IL-6 was defined as the highest IL-6 concentration recorded within the first 15 days after CAR-T infusion.

**Electrolyte definitions**

Hypophosphatemia was defined as serum phosphate <2.5 mg/dL and categorized as mild (2.0–2.5 mg/dL), moderate (1.0–2.0 mg/dL), or severe (<1.0 mg/dL). Hyponatremia was defined as sodium <135 mmol/L, hypokalemia as potassium <3.5 mmol/L, hypomagnesemia as magnesium <1.7 mg/dL, and hypocalcemia as total calcium <8.5 mg/dL. Severity categories for electrolyte disturbances were defined according to prespecified biochemical thresholds.

**Hematological definitions**

Progression-free survival (PFS) was defined as the time from CAR-T infusion to relapse, progression, or death from any cause. Disease status before CAR-T therapy was classified as: i) primary refractory disease, in patients who never achieved end-of-treatment response; or ii) prior chemosensitivity, in patients who responded to previous treatment but subsequently experienced relapsed or refractory disease before CAR-T therapy.

**Urinary indices**

Fractional excretion of phosphate was calculated using the following formula:

FEPO₄ (%) = [(urine phosphate × serum creatinine) / (serum phosphate × urine creatinine)] × 100.

FEPO₄ >5% was considered suggestive of inappropriate renal phosphate loss in the context of hypophosphatemia. However, because FEPO₄ depends on serum phosphate concentrations, it may be affected by rapid changes in serum phosphate levels or intracellular phosphate redistribution. Therefore, FEPO₄ was interpreted as an exploratory index of renal phosphate handling rather than definitive proof of renal phosphate wasting.

Urinary protein excretion was assessed using urine protein-to-creatinine ratio (uPCR), expressed in g/g. Urinary alpha-1-microglobulin-to-creatinine ratio (uA1M/Cr) was used as an additional urinary marker compatible with tubular dysfunction. Urine dipstick testing was reviewed for glycosuria.

**Statistical analyses**

Categorical variables were expressed as numbers and percentages, and continuous variables as mean ± SD or median with interquartile range (IQR), as appropriate. The normal distribution assumption was assessed using the Shapiro–Wilk test. Between-group differences were evaluated using Student’s t-test or the Mann–Whitney U test for quantitative variables, and the chi-square test or Fisher’s exact test for categorical variables. Correlations between continuous variables were assessed using Spearman’s rank correlation coefficient. Exploratory partial Spearman correlation analyses were performed to assess the association of phosphate nadir with inflammatory, urinary, and mineral metabolism parameters after adjustment for selected covariates. Given the limited sample size, these analyses were considered exploratory and hypothesis-generating.

Multivariable logistic regression was used to assess variables associated with moderate hypophosphatemia. Given the limited sample size and number of events, the model was intentionally exploratory. Variables were selected based on biological plausibility and univariable associations. Survival analyses were performed using Kaplan–Meier curves and Cox proportional hazards models. Exploratory analyses of PTH were additionally performed after excluding patients with baseline eGFR <60 mL/min/1.73 m². A two-sided p-value <0.05 was considered statistically significant. Statistical analyses were conducted using SPSS Statistics version 25 and RStudio.

**SUPPLEMENTARY RESULTS**

Table S1. Electrolyte values according to phosphorus levels.

|  | N = 63 | Phosphorus nadir levels (mg/dL) | | P value |
| --- | --- | --- | --- | --- |
|  |  | $<$ 2 (n=26) | $\geq2$ (n=37) |  |
| Sodium nadir in mmol/L | 134 (132-135) | 132 (130-135) | 134 (133-136) | 0.05 |
| Potassium nadir in mmol/L | 3.3 (3.2-3.5) | 3.3 (3.0-3.4) | 3.4 (3.2-3.6) | **0.04** |
| Magnesium nadir in mg/dL | 1.6 (1.5-1.8) | 1.6 (1.5-1.7) | 1.6 (1.6-1.8) | 0.27 |
| Calcium at day 7 in mg/dL | 9.1 (8.7-9.4) | 8.9 (8.4-9.4) | 9.2 (8.9-9.4) | 0.20 |

Values are expressed as median (interquartile range, IQR))

Figure S1. Correlation between changes in log-transformed FGF23 and phosphate levels between baseline and day +7 following CAR-T therapy. No significant association was observed (Spearman ρ = 0.05, p = 0.72).

Figure S2. Log-transformed FGF23 levels at baseline (day 0) and day +7 after CAR-T infusion according to hypophosphatemia severity. Patients are stratified by phosphate nadir ≥2 mg/dL and <2 mg/dL. Points represent individual patients; horizontal bars indicate mean values with 95% confidence intervals.

Figure S3. Correlation between parathyroid hormone (PTH) and phosphate nadir following CAR-T therapy. Higher PTH was associated with lower phosphate nadir (Spearman ρ = −0.26, p = 0.045).

Figure S4. Correlation between urine potassium-to-creatinine ratio (CoKCr) and potassium nadir following CAR-T therapy. CoKCr values are displayed on a log10 scale. Higher urinary potassium excretion was associated with lower potassium nadir (Spearman ρ = −0.35, p = 0.006).

REFERENCES

1.	Mirzazadeh M, Webster C, Weerasinghe G, Morris T, James T, Shine B. UK Reference Intervals for Parathyroid Hormone Using Abbott Methods. Br J Biomed Sci. 2023 Apr 17;80:11224. doi:10.3389/bjbs.2023.11224 PubMed PMID: 37139470; PubMed Central PMCID: PMC10150696.
